# Perceptions of User-Generated Content as a Source of Health Messages in Smoking Cessation Mobile Interventions: Focus Group Study

**DOI:** 10.2196/76804

**Published:** 2025-12-17

**Authors:** Michael Wakeman, Lydia Tesfaye, Tim Gregory, Erin Leahy, Gunnar Baskin, Greg Gruse, Brandon Kendrick, Sherine El-Toukhy

**Affiliations:** 1 Division of Intramural Research, National Institute on Minority Health and Health Disparities, 11545 Rockville Pike, Rockville, MD, 20852, United States, 1 3015944743; 2 Pritzker School of Medicine, University of Chicago, Chicago, IL, United States; 3 School of Medicine, Emory University, Atlanta, GA, United States; 4 ICF NEXT (United States), Reston, VA, United States

**Keywords:** focus group discussions, low socioeconomic status, smoking cessation apps, young adults, user-generated content, social media, Twitter, X

## Abstract

**Background:**

Health messages are integral to smoking cessation interventions. Common approaches to health message development include expert-crafted messages and audience-generated messages, which produce messages that can be monotonic, didactic, and limited in number. We introduce an alternative approach to health message development that relies on user-generated content available on open-content platforms as a source of health messages.

**Objective:**

We examined the acceptability of user-generated content curated from Twitter (subsequently rebranded X) as a source of health support messages in a newly developed smoking cessation mobile intervention called Quit Journey and the optimal timing and frequency with which health messages can be deployed to support app users in real time.

**Methods:**

A total of 12 semistructured focus groups were held with 38 young adults with low socioeconomic status who smoked cigarettes, wanted to quit, and were aged 18 to 29 years. Focus groups were held virtually on GoTo Meeting, audio recorded, and transcribed verbatim. Deductive thematic analysis was used, with themes based on 5 constructs from the second unified theory of acceptance and use of technology (ie, effort expectancy, facilitating conditions, hedonic motivation, performance expectancy, and social influence) and negative, neutral, and positive sentiment.

**Results:**

Participants perceived user-generated content positively (56/108, 51.9% of the quotes) and focused on their perceived usefulness (37/108, 34.3% of the quotes). User-generated content was perceived as authentic, nonrepetitive support from people with similar real-life experiences. Negative or sarcastic user-generated content elicited negative reactions from participants. Participants preferred receiving 3 or fewer daily messages, ideally before cravings. Suggestions focused on the need to screen user-generated content before its inclusion in the app library and allow app users to customize message frequency and timing.

**Conclusions:**

User-generated content was deemed an acceptable source of health messages. This content can improve the efficacy and effectiveness of smoking cessation interventions by increasing their pool of unique messages that may be better received and more persuasive than expert-curated content. User-generated content can be used to curate health messages for all medical conditions and behaviors with relevant publicly available online content for integration in behavioral interventions given its high volume, brevity, and narrative-like nature. Future research is needed to investigate the effects of user-generated content on health behaviors and identify the theoretical mechanisms for these effects.

## Introduction

Health messaging is integral to smoking cessation efforts, including behavioral interventions [1,2]. Effective health messages are understandable; informative; engaging; and, most importantly, capable of inducing behavior change [3]. Despite the importance of health messages to behavioral interventions, the process of health message development is “commonly hidden within a black box” [4]. The most common is a top-down approach to message development, where interventions include expert-crafted health messages that embody clinical or expert guidelines, determinants of behavior change as outlined in theoretical frameworks (eg, health belief model, theory of planned behavior, and elaboration likelihood model), message tactics (eg, gain framed and narratives), and approaches (eg, targeting and tailoring) [3]. Alternatively, target populations have been involved in developing health messages in a bottom-up approach, although this approach is less frequently used [5]. We propose a different approach, where health messages are curated from online open-content sources for use in smoking cessation interventions.

There are limitations to current approaches to health message development. Expert-constructed messages can be perceived as dry, didactic, or unrelatable and can be met with persuasion resistance from audiences [6,7]. Target population–constructed messages can be difficult to generate, especially at scale. Both approaches to message development generate a finite number of messages that are recycled over the course of an intervention. Message repetitiveness can result in message fatigue, which leads to unfavorable message attitudes and reduced message effectiveness, credibility, and behavioral intentions [8,9]. This is particularly important given the need for a substantial pool of candidate messages to cover the extended treatment times of smoking cessation interventions, which usually last 4 to 6 weeks (and often require multiple quit attempts), and the need for personalized message content and intensity under adaptive interventions [10,11].

Web 2.0 and social media platforms have an endless reservoir of user-generated health content owing to their active users, including 2 billion for Instagram and 600 million for Twitter (subsequently rebranded X) [12]. User-generated content is defined as “content [that] is created or produced by the general public rather than by paid professionals and primarily distributed on the internet” [13]. There is evidence suggesting that people turn to online platforms, including social media, for health purposes. For instance, health discussion forums, social media platforms, and crowdsourcing platforms have user-generated smoking cessation content [14,15]. In 2018, a total of 70.14% of Americans used digital means to access health information, whereas 14.02% shared health information via social media platforms [16]. However, research on Web 2.0 and social media platforms has been limited to examining tobacco advertising and audience-targeting strategies and their associations with tobacco use behaviors [17-19]; analyzing opinions and attitudes regarding tobacco products and the viability of predicting user behavior based on online media posts [20-23]; and, finally, engaging with and disseminating antitobacco messaging and cessation interventions [14,24-26].

User-generated content remains a practically untapped resource for persuasive health messaging. There is evidence suggesting that users trust content created by those going through similar experiences [27]. User-generated online content is also akin to unscripted peer-to-peer communication, which is typically conversational and can include narrative elements or storytelling. These content characteristics can induce behavior change through various mechanisms, such as reducing counterarguments and facilitating role-modeling of the behavior [28-31]. For example, peer messages have been associated with greater engagement with tobacco interventions compared to expert messages [32], whereas emotional and personal testimonials have been associated with greater quitting among individuals with low and middle socioeconomic status (SES) who smoke than among those with high SES [33].

In this study, we explored reactions to the potential of user-generated content as a source of support messages in a smoking cessation intervention [34] called Quit Journey that targets individuals with low SES who smoke cigarettes. The app will feature an on-demand message library and just-in-time support messages to be sent to users when they are at risk of a lapse or relapse. These messages will be drawn from Twitter, a microblogging platform. As target audiences’ reactions to health messages are critical determinants of their effectiveness [3,35], we gauged the acceptance of the use of Twitter messages for cessation support in Quit Journey among young adults with low SES who smoked and their opinions on the frequency and timing of the just-in-time support messages. This study was part of the preparation phase of the multiphase optimization strategy for the development and evaluation of Quit Journey [36,37].

## Methods

### Ethical Considerations

The National Institutes of Health institutional review board exempted this study on October 11, 2019, under category 2, research that only includes interactions involving educational tests, survey procedures, interview procedures, or observation of public behavior (title 45 of the Code of Federal Regulations, part 46.10(d)(2)), and category 3, research involving benign behavioral interventions (title 45 of the Code of Federal Regulations, part 46.10(d)(3)). ICF International’s institutional review board exempted this study on November 19, 2019, under category 2 and approved an amendment on February 26, 2020. All participants verbally consented to take part in the study. Participants received US $150 as compensation. Participants were assigned numeric identification numbers that were used during the focus group discussions and in the transcripts.

### Participants and Recruitment

We partnered with UserWorks, Inc, to recruit a convenience sample of 38 young adults with low SES who smoked. Individuals with low SES are a priority for smoking cessation efforts, with high prevalence of cigarette smoking among adults with low incomes (18.3%) and those without a high school diploma or who passed the General Educational Development test (20.1% and 30.7%, respectively) [38].

Eligibility criteria included being aged 18 to 29 years; having a low SES, as evidenced by being neither a 4-year college graduate nor a college enrollee [39-41]; being a current smoker who smoked at least 100 cigarettes in their lifetime and reported smoking every day or some days; willingness to quit within 6 months; not currently using any smoking cessation aids or noncigarette combustible tobacco products; being a smartphone owner; and speaking English. Recruitment took place between January 2020 and April 2020. The sample size was deemed sufficient to achieve data saturation, and none of the participants withdrew from the study [42,43]. The 32-item COREQ (Consolidated Criteria for Reporting Qualitative Research) checklist can be found in Table S1 in Multimedia Appendix 1 [44].

### Procedures

We conducted 12 virtual focus groups lasting approximately 1.5 hours each on GoTo Meeting using a topic guide informed by the second unified theory of acceptance and use of technology (Multimedia Appendix 1) [45,46]. In this paper, we focus solely on Quit Journey’s message library and just-in-time support feature, which are under development. The moderator described the concept behind the on-demand message library and just-in-time support feature in 8 focus groups and presented mock app pages with placeholder messages and 5 sample tweets in 4 focus groups. For example, one sample tweet read the following:

*...after seeing a smokers teeth now im glad I haven’t touched a cig in months!!*

All sample tweets can be found in Note S1 in Multimedia Appendix 1. In total, 31.6% (12/38) of the participants were involved in 2 focus groups, one in which the moderator presented the concept behind utilizing user-generated content in Quit Journey and one in which the moderator presented app mock pages with placeholder user-generated content. Our work on perceptions of smoking cessation apps and of Quit Journey and its individual features has been published elsewhere [47-51; Wakeman M, unpublished data, April 2025].

We conducted dry runs to ensure that the sessions ended on time. TG, a male user experience strategist, moderated the discussions. EL, a female strategic communications and marketing project director, was a backup moderator and recorded notes. TG, EL, and SE-T were the only nonparticipants present during the focus group discussions, and none had a previous relationship with the participants. Participants were informed that the moderators were unaffiliated with the research group that commissioned the study. The focus groups were audio recorded and auto-transcribed by GoTo Meeting. Three members of our staff verified these transcripts against the audio files. Transcripts and findings were not returned to participants for feedback.

### Analysis

We adopted a deductive thematic approach to data analysis [52]. After an initial review of the transcripts, we developed codes and corresponding themes based on 5 constructs from the second unified theory of acceptance and use of technology: effort expectancy, facilitating conditions, hedonic motivation, performance expectancy, and social influence [45,46]. Furthermore, we coded the transcripts for negative, neutral, and positive sentiment; design concepts; suggestions for improvement; and Quit Journey app features [49-51]. Briefly, effort and performance expectancies were defined as “perceived ease or effortfulness” and “perceived usefulness or helpfulness” of mobile apps and their features, respectively. Facilitating conditions were defined as “factors that can aid or impede [their] uptake or use,” whereas social influence referred to “perceived importance of significant others’ recommendations and approval” in considering use of mobile apps and their features. Hedonic motivation was defined as “perceived fun, pleasure, or enjoyment (or lack thereof) associated with [their] use.” Negative and positive sentiment captured statements that indicated “a sense of disapproval, criticism, or skepticism” or “a sense of approval, praise, or certainty” about mobile apps, respectively. Neutral sentiment captured remarks that “did not have positive or negative tone, contained equal number of positive- and negative-toned remarks, or were conditional in nature.” The design concept code captured statements related to the visual depiction and aesthetics of mobile apps or their features. Finally, the suggestion code captured statements that were “concerned with improvements, modifications, or adjustments” to mobile apps or their features, whereas the app feature code captured the Quit Journey app feature being discussed (eg, carbon monoxide tracking; Wakeman M, unpublished data, April 2025) [47-51]. Quotes that did not fit under a technology acceptance construct but still conveyed a particular sentiment were coded as “not applicable.”

We introduced new codes if the content in the transcripts did not fit the predefined ones. In coding the transcripts, we applied the predominant theme in each quote even if it was not mentioned explicitly (ie, in response to a moderator’s question). When one theme was contingent on another, we coded the quote for the underpinning theme. We followed a multicoding approach where multiple domains (eg, technology acceptance and sentiment) could be applied to a single quote but only 1 code could be selected from each domain (eg, negative, neutral, or positive).

MW and LT independently coded the focus group transcripts. Using the ATLAS.ti qualitative software (version 8; ATLAS.ti Scientific Software Development GmbH), we calculated 2 intercoder agreement measures: the Krippendorff c-alpha and Krippendorff cu-alpha. The first, a measure of separating relevant and irrelevant content (ie, whether coders identified texts of similar locations and lengths), was 0.82 [53]. The second, a metric related to semantic domain reliability (ie, whether coders coded for the presence or absence of groups of related themes), was 0.66 for technology acceptance, 0.70 for sentiment, and 0.94 for app features [53]. MW, LT, and SE-T discussed discrepancies and resolved them.

## Results

### Overview

Sample characteristics are shown in Table 1, and detailed participant characteristics can be found in Table S2 in Multimedia Appendix 1.

**Table 1.** Participant characteristics (N=38) [47-51].

Characteristics	Participants, n (%)
Sex	
Female	20 (52.6)
Male	18 (47.4)
Race and/or ethnicity	
American Indian or Alaska Native	1 (2.6)
Asian, Native Hawaiian, or Pacific Islander	3 (7.9)
Black or African American	11 (28.9)
Hispanic or Latino	6 (15.8)
White	16 (42.1)
Mixed	1 (2.6)
Highest level of education	
Lower than high school	3 (7.9)
High school graduate	10 (26.3)
High school equivalent	3 (7.9)
Some college, no degree	18 (47.4)
2-year associate degree	4 (10.5)
Smoking frequency	
Every day	30 (78.9)
Some days	8 (21.1)
Quit time frame	
7 days	11 (28.9)
30 days	22 (57.9)
6 months	5 (13.2)
Smartphone operating system	
Android	21 (55.3)
iOS	17 (44.7)

User-generated content was an acceptable source of smoking cessation support messages. Of 108 extracted quotes, roughly half (n=56, 51.9%) reflected a positive sentiment (Table 2). Discussions largely focused on performance expectancy (n=37, 34.3%), followed by effort expectancy (n=7, 6.5%), hedonic motivation (n=7, 6.5%), and facilitating conditions (n=1, 0.9%). All quotes appear verbatim in Tables S3 and S4 in Multimedia Appendix 1.

**Table 2.** Distribution of the number of quotes by technology acceptance and sentiment toward the user-generated message library. Column totals add up to 100% within each theme, whereas overall row totals add up to 100% across a semantic domain (N=108).

Technology acceptance domain	Sentiment, n/N (%)			Total, n/N (%)
	Negative	Neutral	Positive	
Effort expectancy	1/7 (14.3)	0 (0)	6/7 (85.7)	7/108 (6.5)
Facilitating conditions	0 (0)	1/1 (100)	0 (0)	1/108 (0.9)
Hedonic motivation	0 (0)	3/7 (42.9)	4/7 (57.1)	7/108 (6.5)
Performance expectancy	9/37 (24.3)	3/37 (8.1)	25/37 (67.6)	37/108 (34.3)
Social influence	0 (0)	0 (0)	0 (0)	0 (0)
Not applicable	19/56 (33.9)	16/56 (28.6)	21/56 (37.5)	56/108 (51.9)
Total	29/108 (26.9)	23/108 (21.3)	56/108 (51.9)	108/108/ (100)

### Performance Expectancy

Most quotes on the usefulness of user-generated content for smoking cessation (25/37, 67.6%) overlapped with positive sentiment. Reasons for its perceived usefulness included providing nonrepetitive, authentic content from people “going through the same thing as you” (P35). User-generated content was also seen as a validation of the intended audiences’ emotions and experiences while imparting a sense of community and social support:

*I know I get annoyed when...something that’s...giving you motivational quotes and it’ll just start like repeating them because they only have a few*.

[P11]

*Yeah, I feel like it'd be very motivating to have that sense of community [from the tweets] and to see it working in real-time and kind of have that push to be inspired to keep going, if you see actual people getting benefits from it*.

[P10]

Participants emphasized the benefits of knowing the experiences of others, which could improve their self-efficacy and motivation to quit:

*I think [the tweets] would be super helpful for people.... The community who have ended up quitting smoking, I think it would be super helpful to see what they have to say and what they think about it after the fact. So, yeah, I like that a lot*.

[P08]

*I think it’s nice to have a personal touch and [the tweet] gives the user a reminder that it’s possible to achieve the outcome you're wanting and...there’s proof from other people that have been able to overcome the...addiction that we're all trying to kick.... There are key points that I can see in some of these [example tweets] that I'm thinking to myself are applicable to me and concerns that I have. So, it’s just another reminder and incentive and...positive reinforcement that I think is a great idea*.

[P14]

Quotes with a negative sentiment (9/37, 24.3%) captured skepticism about the relevance of others’ success stories or their motivating impact on individual participants’ quit journeys:

*I just don't think that...reading what other people are doing...is like helpful to me and...if I'm quitting smoking, I don't care if someone else...is a “proud owner of a tobacco free body,” right. Like, there’s lots of people who don't smoke. But...that’s not my journey*.

[P11]

Negative sentiment also reflected participants’ reactions to sample tweets that were perceived as sarcastic or ironic in tone or those that referenced the adverse health effects of smoking:

*I don't like the first [example tweet], because I feel like that would just make me feel bad, especially if like my teeth are already yellow from smoking.... If you’re...struggling and then someone else is, like, “Oh, yay.” Like, you know, “I'm doing so well and blah blah blah,” and...that can make you feel kind of...bad. And then...if you feel that you might just start smoking more because you're like, what’s the point, I’m a failure*.

[P11]

### Other Technology Acceptance Themes

Participants perceived Quit Journey’s tweet-based message library as both easy and fun to use. Quotes reflecting effort expectancy, or the perceived ease of using the message library, and hedonic motivation, or the perceived enjoyment of using the message library, were generally positive (6/7, 85.7% and 4/7, 57.1%, respectively):

*I think it will be fun [to include tweets]*.

[P16]

Discussions rarely focused on conditions that could facilitate or impede the use of a Twitter-based message library or just-in-time support messages. For example, one participant had no privacy concerns, noting that they would be comfortable with the app collecting their ratings of messages to personalize message content as the app learned their preferences.

### Suggestions

An additional 105 quotes focused on participants’ suggestions related to message content, timing, and frequency. First, to maintain the authenticity of user-generated content (ie, ensuring that app users know that the messages were authored by real people), participants suggested that messages be attributed to the poster’s name, Twitter handle, or online platform. Others suggested that the app’s library maintain the visual presentation of Twitter messages to signal the messages’ authenticity. Finally, participants suggested that the tweets should be unedited or unaltered, including emojis and any spelling or grammatical errors unless they jeopardized the meaning of the message. If edited, it should be minimal to aid comprehension:

*If you're going to have [tweets], yeah, it’s better to know that it’s from a real person than like a computer-generated thing*.

[P11]

*Just mention the name of the platform the messages are from but exclude the name of the people who posted. Keep them anonymous*.

[P06]

*We use social media a lot, we do a lot of misspelling, we miss punctuations, and all of that stuff. So...if that is gonna be coming from an actual person...you want to show...not just the good, but the ugly too [in the tweets], you know not everyone can spell*.

[P16]

Participants emphasized that sarcastic, ironic, or negative tweets should not be included in the app’s message library:

*I would just be cautious with what [Twitter] messages you use.... But I like the idea, like, getting a notification. Maybe...every day or twice a day or something that has something kind of inspirational to help you. But...I think it’s...really a matter of choosing ones...that are all positive*.

[P05]

Regarding push messages, participants suggested tailoring the messages based on age, smoking behavior, and ratings of previous messages, among other factors. Furthermore, participants emphasized the importance of being able to customize the timing and frequency of notifications. For example, although feedback was mixed, most participants preferred to receive support messages before a craving occurred. Participants suggested that they would be receptive to 1 to 10 messages per day, with 3 messages per day being the most common:

*I guess [I would prefer to receive messages] before, or during the craving, because if it comes afterwards, then you probably already smoked one*.

[P10]

*So, in my opinion, it would be that if you're making a really good progress, the less [tweets] that you may need, or it can be the opposite...if you're doing really good, more [tweets].... I don't know. I think maybe start off three a day—morning, noon, night*.

[P16]

*I feel like [the tweets are] really personal and probably even could like learn me in a way where if I’m having...a bad day or...there’s certain times where I feel like I need a little more support then.... Just to kind of like throw it at me and then if I have to change it then I will change it, you know?*

[P02]

## Discussion

### Principal Findings

This study showed that user-generated content was an acceptable source of support messages in Quit Journey, a smoking cessation intervention targeting individuals with low SES who smoke. The perceived usefulness of user-generated content reflected core characteristics of effective health messaging, including their brevity, relatedness to target populations, and narrative-like nature, while avoiding pitfalls common to expert-designed messages, such as monotony, repetitiveness, and didacticism [3,6]. Suggestions to improve user-generated content for use in Quit Journey’s message library and just-in-time support feature included refraining from overly negative or sarcastically toned messages and tailoring the messages to users’ characteristics, time-varying needs, and message preferences [3]. Cumulatively, these results suggest that we can leverage user-generated content to build Quit Journey’s message library. More broadly, user-generated content marks a departure from the limited number of top-down, expert-designed messages commonly used in health interventions, which can be subject to persuasion resistance and message fatigue [8,9]. Moreover, the use of user-generated content exponentially increases the pool of candidate messages, which allows for the tailoring of message content, frequency, and timing to users’ needs. This is particularly true for technology-based smoking cessation interventions, where the capture of time-varying psychological, physiological, and contextual factors is increasingly feasible [54,55]. Our proposed approach of relying on user-generated content is applicable to practically all health conditions and behaviors with relevant publicly available online content, including addictive and lifestyle behaviors [56,57].

### Comparison to Prior Work

User-generated content includes elements of effective health messages, including its brevity and narrative-like nature, which can improve message persuasiveness and effectiveness in promoting behavior change [30,31]. Indeed, our participants attributed the usefulness of user-generated content to its realism and role-modeling from others who have attempted to quit, successfully or otherwise. For example, one participant noted their aversion to “computer-generated” messages (P11), whereas another noted how these messages could improve self-efficacy, saying that “if someone else can do it, maybe...you can do it too” (P21). These positive perceptions may also reflect involvement with message creators and trust in the health information provided as personal stories by those perceived to be similar to them and with firsthand experiences [27,29,58]. Evidence shows that peer messages and emotional and narrative content have been used to effectively treat nicotine dependence and are associated with greater engagement with tobacco cessation interventions [32,59]. Notably, tweets are similar in length to SMS text messages, which have proven successful as smoking cessation interventions [60]. While user-generated content has been shown to influence nonhealth decision-making (eg, purchasing decisions) [61,62], future research should examine its persuasive effect on health behaviors, identify mechanisms of its effect (eg, self-efficacy and perceived norms), and compare its effectiveness to other content generation approaches (eg, expert-crafted messages and artificial intelligence conversational agents or chatbots [63]).

Open-source content negates message fatigue associated with message repetition [8,9]. A typical smoking cessation intervention is 6 to 8 weeks long and includes a fixed number of support messages (eg, the National Cancer Institute’s SMS text messaging intervention SmokefreeTXT) [64]. An intervention recipient is bound to receive the same messages during the intervention once they have exhausted the number of messages dedicated to a particular purpose (eg, craving support). Furthermore, individuals who smoke often attempt to quit several times before they are successful [65]. This means that they will be exposed to the same messages if they reset their quit day or re-enroll in an intervention. Additionally, tailoring messages to individuals’ characteristics and time-varying factors as they undergo an intervention quickly becomes a challenge [66]. This could be one of the reasons why many smoking cessation interventions use only basic tailoring strategies (eg, addressing an intervention recipient by their name) [67]. A large pool of candidate smoking-related messages curated from Twitter or other platforms can facilitate tailoring of intervention message content.

Our results show that the appeal of user-generated content as a source of cessation support messages was dependent on message content and attributes. Participants generally preferred positive messages over those perceived as sarcastic or ironic, consistent with previous research [68]. Furthermore, perceptions were mixed on messages that focused on the adverse health effects of smoking. These discussions reflect debates on the use of fear appeals (ie, messages that include a threat) and gain- versus loss-framed messages (ie, messages that emphasize benefits of compliance with message recommendations or losses associated with noncompliance, respectively) in health interventions [69,70]. There is evidence suggesting that fear appeals can be effective in promoting smoking cessation [71-73], including among individuals with low SES who smoke [2]. It is noteworthy that the persuasiveness of a message depends on various factors, such as the behavior in question (eg, one time vs repeated and prevention vs detection), audience characteristics (eg, readiness for change), and other message elements (eg, inclusion of self-efficacy statements). For example, evidence shows that gain-framed messages can be slightly more effective in promoting some preventive behaviors, including smoking cessation [74,75]. Additionally, there are unintended and ethical consequences of fear appeals and loss-framed messages and questions about their long-term effects on behavior [76]. Taken together, feedback from our participants and existing literature suggests a need to identify the underlying theoretical concepts conveyed in user-generated messages and investigate the effect of different types of user-generated messages on behavioral outcomes at various stages of a smoking cessation intervention.

Participants’ suggestions centered on message attribution and content and the frequency and timing of push messages. They emphasized the appeal of attributing the messages in Quit Journey to their actual authors. This is consistent with research showing that message source can increase trust and operate as a cue to persuasion and attitude change [27,77]. Relatedly, participants noted the need for the messages to be authentic and human-authored, suggesting that the tweets retain their conversational tones and even grammatical errors. Finally, participants emphasized the need to tailor message content to personal and dynamic factors [66,78,79]. For instance, participants suggested several tailoring factors, including cravings, mood, and stage of quitting. Furthermore, participants suggested that, on average, they would be open to receiving up to 3 daily push notifications but preferred to have the ability to customize the frequency and timing of these messages. While these requests are common [80], they defeat the purpose of just-in-time support [10]. A compromise could be to allow users some control over the number of daily messages they want to receive and giving them options to customize other aspects of the intervention (eg, app aesthetics).

### Strengths and Limitations

To our knowledge, our approach to curating publicly available user-generated content in a smoking cessation mobile intervention (and in health interventions generally) is the first of its kind [22,54]. In this study, we demonstrated initial acceptance of this approach among the target population of our smoking cessation intervention, Quit Journey. Study strengths include a diverse group of participants, with no more than 50% being from one race and/or ethnicity. Limitations include the reliance on educational level as a sole indicator for SES [39-41]. The focus groups were held virtually due to the COVID-19 pandemic, which may have impacted engagement in discussions. Additionally, the pandemic had mixed effects on smoking behaviors [81], which consequently could have affected participants’ perceptions of our smoking cessation mobile app and its components, including the proposed user-generated message library to support smoking cessation. Participants were only shown 5 example tweets, which were not representative of the wide range of smoking-related tweets that could have elicited different reactions from participants. Furthermore, because the message library and just-in-time support features of Quit Journey were still under development, participants were presented with mock pages with placeholder messages. Participants were not representative of all individuals who smoke, such as older individuals, some of whom may not be as receptive to social media content as the young adults in our study [82]. Finally, due to scheduling conflicts, focus groups had unequal numbers of participants.

### Conclusions

This study provided evidence of the acceptability of user-generated content as a source of support messages in smoking cessation interventions. Implementing this new approach to developing health messages requires additional research to select and evaluate user-generated content with the target audience before these messages are integrated into a behavioral intervention. Although we focused on smoking cessation, our proposed approach to health message development can be applied to other health conditions and risky behaviors to accommodate increasing needs for content-diverse, relatable, and short messages that can support individual users and their dynamic needs during behavior change.

## Supplementary material

10.2196/76804 Multimedia Appendix 1 Themes and illustrative quotes of participants’ perceptions of performance for a Twitter/X message-based library and illustrative quotes of participants’ suggestions for improving a Twitter/X message-based library.

## Abbreviations

COREQ: Consolidated Criteria for Reporting Qualitative Research
SES: socioeconomic status
