# Supplementary material for: Perceptions of User-Generated Content as a Source of Health Messages in Smoking Cessation Mobile Interventions: Focus Group Study
Source: JMIR Hum Factors. 2025 Dec 17;12:e76804. doi: 10.2196/76804 (PMC12711135; doi:10.2196/76804)
Supplement: Multimedia Appendix 1 [file humanfactors-v12-e76804-s001.docx]

**Supplementary Table 1. Consolidated criteria for reporting qualitative studies (COREQ)** [44].

| Topic | Guide Questions/Description | Page No. |
| --- | --- | --- |
|  |  |  |
| Domain 1: Research team and reflexivity |  |  |
|  |  |  |
| *Personal Characteristics* |  |  |
| Interviewer/facilitator | Which author/s conducted the interview or focus group? | 5 |
| Credentials | What were the researcher’s credentials? | 5 |
| Occupation | What was their occupation at the time of the study? | 5 |
| Gender | Was the researcher male or female? | 5 |
| Experience and training | What experience or training did the researcher have? | 5 |
|  |  |  |
| *Relationship with participants* |  |  |
| Relationships established | Was a relationship established prior to study commencement? | 5 |
| Participant knowledge of the interviewer | What did the participants know about the researcher? | 5 |
| Interviewer characteristics | What characteristics were reported about the interviewer/facilitator? | 5 |
|  |  |  |
| Domain 2: Study design |  |  |
|  |  |  |
| *Theoretical framework* |  |  |
| Methodological orientation and Theory | What methodological orientation was stated to underpin the study? | 5 |
|  |  |  |
| *Participant selection* |  |  |
| Sampling | How were participants selected? | 4 |
| Method of approach | How were participants approached? | 4 |
| Sample size | How many participants were in the study? | 4 |
| Non-participation | How many people refused to participate or dropped out? | 4-5 |
|  |  |  |
| *Setting* |  |  |
| Setting of data collection | Where was the data collected? | 5 |
| Presence of non-participants | Was anyone else present besides the participants and researchers? | 5 |
| Description of sample | What are the important characteristics of the sample? | 6-7 |
|  |  |  |
| *Data collection* |  |  |
| Interview guide | Were questions, prompts, guides provided by the authors? Was it pilot tested? | 5 |
| Repeat interviews | Were repeat interviews carried out? If yes, how many? | 5 |
| Audio/visual recording | Did the research use audio or visual recording to collect the data? | 5 |
| Field notes | Were field notes made during and/or after the interview or focus group? | 5 |
| Duration | What was the duration of the interviews or focus group? | 5 |
| Data saturation | Was data saturation discussed? | 4 |
| Transcripts returned | Were transcripts returned to participants for comment and/or correction? | 5 |
|  |  |  |
| Domain 3: Analysis and findings |  |  |
|  |  |  |
| *Data analysis* |  |  |
| Number of data coders | How many data coders coded the data? | 6 |
| Description of the coding tree | Did authors provide a description of the coding tree? | 5-6 |
| Derivation of themes | Were themes identified in advance or derived from the data? | 5 |
| Software | What software, if applicable, was used to manage the data? | 6 |
| Participant checking | Did participants provide feedback on the findings? | 5 |
|  |  |  |
| *Reporting* |  |  |
| Quotations presented | Were participant quotations presented to illustrate the themes/findings? Was each quotation identified? | 7-9 |
| Data and findings consistent | Was there consistency between the data presented and the findings? | 7-9 |
| Clarity of major themes | Were major themes clearly presented in the findings? | 7-9 |
| Clarity of minor themes | Is there a description of diverse cases or discussion of minor themes? | 7-9 |

**Supplementary note 1. Moderation guide.**

**Introduction.**

The sponsors of this focus group developed Quit Journey, a smoking cessation mobile application, to help those who want to quit smoking. I’d like to describe features of this app and get your reactions.

The app has a library with quotes from people who have quit smoking for motivation, to give smokers reasons to quit, and to inform them of different methods to deal with cravings and stress. There are many sources of messages and stories people put online about quitting. We used messages from Twitter because they are short, and we think they are relevant to someone who wants to quit. For example, people wrote:

1. *‘after seeing a smokers teeth now im glad I haven’t touched a cig in months!!!’*
2. *‘got a Nicorette inhaler its amazing! Haven’t had a cig all day’*
3. *‘proud owner of a tobacco free body!’*
4. *‘every time I see someone smoking a cig I just think there’s someone I can outlast when the world ends’*
5. *‘every time you want to take a cig carry around your favorite candy and eat that instead’*

**Questions**

1. What is your general reaction to this idea? Is there anything you especially like about it? Is there anything you especially dislike? Is anything confusing?
2. What do you think of these app’s screenshots? What do you suggest to improve them?
3. What do you like about it? What do you dislike about it? What do you suggest to improve it? How useful would the app be to you with these features? Do you think the app with these features would be fun to use? Do you think the app with these features would be easy to use?

**Supplementary** **Table 2. Participant characteristics** [47-51].

| **ID** | **Sex** | **Race and ethnicity** | **Highest level of education** | **Smoking frequency** | **Quit timeframe** | **Smartphone operating system** |
| --- | --- | --- | --- | --- | --- | --- |
|  |  |  |  |  |  |  |
| P01^*^ | Female | White | Some college, ND | Every day | 30 days | Android |
| P02^*^ | Male | Hispanic/Latino | Some college, ND | Every day | 30 days | Android |
| P03 | Female | Hispanic/Latino | HS incomplete | Every day | 7 days | Android |
| P04^*^ | Female | White | Some college, ND | Some days | 30 days | Android |
| P05 | Male | White | Some college, ND | Every day | 7 days | Android |
| P06 | Male | Black/AA | Some college, ND | Every day | 30 days | Android |
| P07 | Male | Asian, NHPI | HS equivalent | Every day | 30 days | iOS |
| P08^*^ | Female | Hispanic/Latino | Some college, ND | Every day | 30 days | iOS |
| P09 | Male | White | HS equivalent | Every day | 30 days | iOS |
| P10^*^ | Male | White | HS graduate | Every day | 7 days | Android |
| P11^*^ | Female | Hispanic/Latino | Some college, ND | Every day | 30 days | iOS |
| P12^*^ | Male | White | Some college, ND | Every day | 7 days | Android |
| P13^*^ | Female | White | HS graduate | Every day | 30 days | Android |
| P14^*^ | Female | White | Two-year AD | Some days | 30 days | iOS |
| P15^*^ | Female | Black/AA | HS incomplete | Some days | 30 days | Android |
| P16^*^ | Female | AIAN | HS incomplete | Every day | 30 days | iOS |
| P17^*^ | Male | Black/AA | HS graduate | Every day | 30 days | Android |
| P18 | Female | Black/AA | Some college, ND | Every day | 7 days | iOS |
| P19 | Female | White | HS equivalent | Every day | 7 days | Android |
| P20 | Female | White | HS graduate | Every day | 7 days | Android |
| P21 | Female | White | Some college, ND | Every day | 7 days | iOS |
| P22 | Female | White | Some college, ND | Every day | 30 days | iOS |
| P23 | Male | White | Some college, ND | Every day | 7 days | iOS |
| P24 | Female | Black/AA | Some college, ND | Every day | 30 days | Android |
| P25 | Female | Black/AA | HS graduate | Some days | 30 days | Android |
| P26 | Male | Black/AA | Some college, ND | Every day | 30 days | iOS |
| P27 | Male | Hispanic/Latino | Some college, ND | Some days | 30 days | Android |
| P28 | Female | White | Two-year AD | Every day | 30 days | Android |
| P29 | Female | White | Some college, ND | Every day | 30 days | iOS |
| P30 | Male | Asian, NHPI | HS graduate | Some days | 6 months | Android |
| P31 | Male | Black/AA | Some college, ND | Every day | 6 months | Android |
| P32 | Female | Asian, NHPI | HS graduate | Every day | 30 days | iOS |
| P33 | Male | Hispanic/Latino | Two-year AD | Some days | 6 months | iOS |
| P34 | Male | Black/AA | Two-year AD | Every day | 6 months | Android |
| P35 | Female | Black/AA | HS graduate | Every day | 30 days | iOS |
| P36 | Male | White | Some college, ND | Every day | 7 days | Android |
| P37 | Male | Black/AA | HS graduate | Every day | 6 months | iOS |
| P38 | Male | Mixed | HS graduate | Some days | 7 days | iOS |

AA= African American, NHPI = Native Hawaiian/Pacific Islander, AIAN= American Indian/Alaska Native
HS = high school, ND = no degree, AD = associate degree

Participants who identified as Hispanics or Latinos were considered as such regardless of race.

^*^ Participated in two focus groups.

**Supplementary Table 3.** **Themes and illustrative quotes of participants’ perceptions of performance for a Twitter/X message-based library.**

| **Themes** | **Quotations** | **Sentiment** |
| --- | --- | --- |
| Performance expectancy | P11: I don't like the first [example tweet], because I feel like that would just make me feel bad, especially if like my teeth are already yellow from smoking … If you're … struggling and then someone else is, like, “Oh, yay.” Like, you know, “I'm doing so well and blah blah blah,” and … that can make you feel kind of … bad. And then … if you feel that you might just start smoking more because you're like, what's the point, I’m a failure. | Negative |
|  | P36: If you guys are actually going to use [tweets] like “A proud owner, a tobacco free body,” is definitely cheesy, but … I don't know that could be motivating for some people. Not me. | Negative |
|  | P33: I get what they're doing with [the message page]. But just for me personally … that’s weird. It does nothing.^1^ | Negative |
|  | P13: I don’t think little sentences are … all that helpful anyways. Like, usually if I'm reading something to make a change, I like reading … full posts from somebody and … they're actually explaining it … That’s more helpful than … just little sentences. | Negative |
|  | P11: Some of [these tweets] could … make you feel bad. Like, if … I had a cigarette yesterday or … today then you read someone that’s like, oh, I haven't had a cigarette all day … Or like if you have like really yellow … bad teeth … that's just going to make you feel bad. And I don't think … other people's information can all the time be … helpful for you. | Negative |
|  | P11: I usually don't like that kind of stuff [like the tweets] … cause … if I don't relate to it then it’s just kind of … unhelpful, or sometimes … people can be … overly positive or … push certain products and I just think … it’s … not something I particularly enjoy. | Negative |
|  | P11: I just don't think that … reading what other people are doing … is like helpful to me and … if I'm quitting smoking, I don't care if someone else … is a “proud owner of a tobacco free body,” right. Like, there's lots of people who don't smoke. But … that's not my journey. | Negative |
|  | P13: I don't think tweets are that useful … if I really wanted to go … look at tweets, I just go on Twitter … I find it more discouraging. | Negative |
|  | P11: [The tweets would] not [be] useful at all. | Negative |
|  | P15: I don't know … [the usefulness] depends. For some people, you know [the tweets] might be good, for others it might not, so I don't know. I don't really have an opinion on it. | Neutral |
|  | P12: I don’t know how useful [the tweets] would be to me. | Neutral |
|  | P22: I like … the fifth [tweet] … That's like helpful. But then … something like [the third tweet], “proud owner of a tobacco free body,” I don't know, that doesn't really help me or make me feel better. Like, oh, good for you. I'm still smoking. It doesn’t make me feel like, yeah, I can do this … I'm not there yet. | Neutral |
|  | P10: Yeah, I feel like it'd be very motivating to have that sense of community [from the tweets] and to see it working in real-time and kind of have that push to be inspired to keep going, if you see actual people getting benefits from it. | Positive |
|  | P08: I think it would be nice to get somebody's perspective on things and their opinion, so you know someone … feels the same way that you do. | Positive |
|  | P27: I think [the tweets] can be very helpful and supportive. | Positive |
|  | P17: [The tweets] motivates you to … stop smoking. [I] see the [example tweet] about the teeth. I don’t want my teeth looking crazy. I like my teeth. | Positive |
|  | P14: I think it's nice to have a personal touch and [the tweet] gives the user a reminder that it's possible to achieve the outcome you're wanting and … there's proof from other people that have been able to overcome the … addiction that we're all trying to kick … There are key points that I can see in some of these [example tweets] that I'm thinking to myself are applicable to me and concerns that I have. So, it’s just another reminder and incentive and … positive reinforcement that I think is a great idea. | Positive |
|  | P17: They looking really informing, about, you know, certain comments. It's good to look at [the pages]. | Positive |
|  | P35: I do like the [example tweet about] candy, just giving you tips, because … other people are going through the same thing as you. | Positive |
|  | P03: The fact of people sharing their stories and hearing other people who did it … to put in your head … if they did it, I can do it too, type thing … I like that. I like the whole idea. | Positive |
|  | P15: I like [the tweets], I feel like it's motivation … to help you quit faster. | Positive |
|  | P14: I mean, [the tweet] kind of allows an additional element to provide … motivation and … confirmation to the person that might be struggling that there are other people out there that went through the same exact thing that you're going through, and they've been successful. And … throwing in additional components of all the positive outcomes they personally experienced by sticking through it … I'm just looking at the [first example tweet] and I can think of so many things health wise as well, but it’s like a personal feature that I think is great. | Positive |
|  | P16: I like that … like the first [example tweet] … in mind that's just like giving … a reality check. That's just showing you, like no, that's real life, like a lot of people have bad teeth because they smoked cigarettes, like you can be really healthy … you smoke a cigarette causing … bad breath, messed up teeth … what else? … I like that it sounds like reality, like just in a message, it just gives you a reminder of things that can happen with smoking. So, that's what I like about it. | Positive |
|  | P20: Especially in this day and time there's a lot … where people feel as if … they’re alone, and they’re the only ones in the world that’s going through … whatever issue they may be going through at this time. We live in a day and time now that people are actually starting to use their voice, and I think it's … really important that we take advantage of that for positive reasons, as this. | Positive |
|  | P21: I think [the tweets] would be useful because I feel like with quitting smoking it's hard, it's easy to feel alone. I think that it would be good to see you're not alone … There's plenty of other people who are going through this every single day and … if someone else can do it, maybe, you know, you can do it too. | Positive |
|  | P06: It will be really useful [to receive tweets] considering it can serve as a motivation. | Positive |
|  | P05: [The tweets could help make] you feel, in general, like … you're not the only one. You’re not the only one going through it. | Positive |
|  | P06: The [most useful new feature is the Twitter] messages … Just getting to know that I’m not alone. | Positive |
|  | P11: I think just information or tips and tricks [in the tweets] is helpful. | Positive |
|  | P23: I think [tweets would be] really pretty useful because it's nice to relate to people and … you're going through something and … you see that … other people are also … feeling the same thing … Obviously, there's like millions of people that smoke cigarettes, but … sometimes you're just like … oh, this … stuff is killing me, and you just … need to kinda feel like there's other people that are … also dying of the same thing. | Positive |
|  | P09: [The tweets would be] very useful. | Positive |
|  | P10: Yeah, I think [the tweets would be] very useful as well. | Positive |
|  | P07: I think [the tweets] would be very useful. | Positive |
|  | P10: I'm looking at the quotes now … and I think it could be helpful to kinda have … a sense of community, because I feel like … it's easier to quit with a sense of community and … like you're not alone in it, so I thought the quotes could be kinda motivational in that way. | Positive |
|  | P09: I definitely agree, some of those quotes are definitely hitting home because I smoke on a daily basis, and … I know how people perceive me as a smoker, and it’s absolutely disgusting. So, quotes of determination and stuff from other people, that'd be awesome. | Positive |
|  | P08: I think [the tweets] would be super helpful for people … The community who have ended up quitting smoking, I think it would be super helpful to see what they have to say and what they think about it after the fact. So, yeah, I like that a lot. | Positive |
|  | P17: Yes, yeah, [the tweets] would [motivate me]. | Positive |
| Effort expectancy | P14: I feel like looking at this [message page] visually I wouldn’t like it, and I would be one of those users who would constantly see something like this pop up and I would always be having a negative reaction … whether that be to snooze it, or remind you later, or just giving it the thumbs down because I feel like it's so much.^1^ | Negative |
|  | P03: I like [being able to rate the tweets]. I think it’s very convenient. | Positive |
|  | P14: As far as notifications within phone apps, I feel like some are very simple and great … In this case, it just being a positive [tweet] is so simple. | Positive |
|  | P05: Oh, definitely [the tweets seems easy to use]. It seems like it would be automated. | Positive |
|  | P04: I like the snooze button idea [for the tweets] because sometimes … if you're getting busy and you actually do want to read these? Save it for later. | Positive |
|  | P23: [The tweets look] pretty easy to use. | Positive |
|  | P10: I think so, yes [it would be easy to use the tweets]. | Positive |
| Hedonic motivation | P02: I think [the tweets] could be [fun to use] … as long as it … feels like it's really engaging, and maybe I would have a successful experience. | Neutral |
|  | P04: Yeah … if you got like an encouraging [tweet] a day or something [it would be fun]. | Neutral |
|  | P11: A robot feeding information can get kind of … boring, but I don't know how to make [the tweets] more personalized, but also … sometimes … what people say is boring too. | Neutral |
|  | P16: I think it will be fun [to include tweets]. | Positive |
|  | P21: I don't really have an exact reason … but I do … think [the tweets] would be enjoyable. | Positive |
|  | P05: Yeah [it would be fun], I'd enjoy that. | Positive |
|  | P09: Most definitely [it would be fun]. | Positive |
| FC | P04: Not really concerned [with the app saving my feedback data]. | Neutral |
| Not applicable | P28: I feel like some of these [example tweets], if this is the kind of stuff that's going to come up, it's a little corny. It reminds me of … what they would tell you in middle school or elementary school to get you to not smoke. | Negative |
|  | P13: If [I] was getting notifications with … tweets, that would be even more annoying. I don't know, I don't really find it that great, Twitter messages. | Negative |
|  | P28: Some of [the example tweets do sound] like a little corny. | Negative |
|  | P30: I really don't think you should go with the first [example tweet]. | Negative |
|  | P33: The more … down to earth phrasing [with the tweets] is … a good idea … It makes it a little corny, but it's also … just ... a little weird to have like … “Hey, did you smoke this week?” Nicole from Omaha, Nebraska on Twitter says, “Tobacco's bad.” It's just weird … it's just kinda odd to me. | Negative |
|  | P37: [The message page is] still a little dead.^1^ | Negative |
|  | P03: I don't like the [first example tweet] … because me personally … I've been smoking for a minute now … I don't have bad teeth … but my mom does. But part of … my mom’s teeth problems isn't because of her smoking cigarettes … so I kinda don't like that quote too much … that just kind of made me feel some type of way about that. | Negative |
|  | P13: I can see this as a really big negative … Like this [example tweet about] bad teeth, or what if … they’re pushing something that's a bad product or makes a person feel bad because they messed up. I don't really like this, actually, the more I think about it. | Negative |
|  | P13: I would want to turn the Twitter stuff off, for sure. | Negative |
|  | P22: I'm still a human being. That [first example tweet is] almost like, oh! I don't want to be a non-smoker like you, you seem like an asshole. | Negative |
|  | P22: Yeah, I feel like if I saw … more than three notifications coming through, and they were … the Twitter posts, and not like check your smoking or anything specifically for me, I’d start getting annoyed and probably just go ahead and turn off notifications. | Negative |
|  | P24: [The rating options] to me, it just looks really plain.^1^ | Negative |
|  | P03: I just don't personally like that, that [first example tweet] in particular. | Negative |
|  | P23: That [first example tweet] is very rude. | Negative |
|  | P10: I don't see the point of having the snooze button neither actually … but if it notifies you anyway, it pops up on your screen and you can see the sentence right there above the snooze button I’d probably just read it anyway before hitting the snooze button anyway. | Negative |
|  | P12: Just thumbs up and thumbs down [to rate the tweets], I kinda just don't like doing that for things because sometimes you see something and ... when it's presented to you with the thumbs up and thumbs down, that's … kind of overly simplistic way of looking at ideas, that it's either good or bad, or … I like it or I don't like it. Sometimes I feel like in-between, and I don't want to … click either one … I'm not saying that there should be more options necessarily, but … I find more and more that … I just get sick of everything … being like thumbs up, thumbs down. | Negative |
|  | P08: The snooze button, I don't really see the point in that. | Negative |
|  | P17: I dislike that idea [of tweets] … It would remind me of … like a commercial … with the puppies on it or … with the people on it that … have their noses cut off because of tobacco … I just wouldn’t like that idea. | Negative |
|  | P34: If you're looking at specific messages … the one [example tweet about] carrying around your favorite candy … you don't want to necessarily replace one habit with another one. | Negative |
|  | P01: I would have to agree that the [tweets] from real people are good. It's just going to be kinda hit or miss with those, because … you might get one … that just doesn't hit home with you … or you might get one that's just exactly what you needed to hear that day, you know, because everybody's different. | Neutral |
|  | P04: I agree that first [tweet example] is a little rough in a way … because people don't want to feel attacked when they're trying to … better themselves … so you want to keep that positive thing flowing. Even the last [example tweet] I enjoy because it's … giving you another out. Like, oh, you want to smoke? Go eat a piece of candy or something or … chew gum … I think the positivity thing is a good thing versus being attacked, because people don’t want to feel attacked. | Neutral |
|  | P11: I was going to say the Twitter messages [would be my least favorite part of the app] if you can't turn it off. I would get really annoyed by that. | Neutral |
|  | P05: I think [tweets are] a good idea … For example, though, the very first message you have there. Personally, I don't have an issue with it … Depending on how it's worded, some people might … take it personally or almost how they feel like maybe an attack. | Neutral |
|  | P02: As long as [the tweet] feels like it’s real. Like, as long as it feels like it's from a real smoker, because the first [example tweet], it doesn't sound like it’s from somebody who has smoked awhile like myself or … is a real smoker. | Neutral |
|  | P11: I don't think I'd want to receive Twitter messages. So, none, but like other inspirational quotes or … information, messages, other kind of stuff, I'd be interested in. | Neutral |
|  | P14: I was actually just thinking about [having the tweets include a source] because I feel like there are so many things out there that are automated now, and advertising sometimes isn't always completely truthful. People can fool and create mock reviews … So, I worry about that. As far as credibility, how accurate is it? Is this truly a real person? Is it some type of computer-related life system, macro code that is just generating all of these things, that are generic sounding? | Neutral |
|  | P09: I don't see the point of having a snooze button really to just see it later, because you can always just go back to it later in your notifications. But I like the idea. | Neutral |
|  | P12: I think it makes it more personal, which could be a good thing. Also, people just say random stuff on Twitter, so that might not be worth looking at. | Neutral |
|  | P12: I liked that [the tweets] could be personalized … not just like a robot that's feeding you information. But … if it's not like a professional opinion, that could be kind of bogus. | Neutral |
|  | P21: I think the last [example tweet] is a horrible, horrible idea … I think that that's just trading … one bad habit for another one. I also think that [example] is kinda shameful … I do like the Nicorette inhaler. I think that's interesting, I actually never heard of that. I like the proud owner of a tobacco free body. I think that's a really positive one … I think it doesn't put anyone else down the way the first one does … [The fourth example] is … kind of funny, and I think that that could be relatable to other people, but … I think that [first] one is … shameful and I think that the last one is just a really bad idea. | Neutral |
|  | P13: I don't really like Twitter's stuff, it's usually just … very small information or very … opinionated stuff and I might not even want to see some of it, you know, like useful information, maybe like the last [example tweet]. Like, oh, “carry around your favorite candy,” or something would be okay, but … maybe there's other stuff I just wouldn't even want to see. | Neutral |
|  | P11: I'm not sure how many … Twitter messages they have. But … I know I get annoyed when … I have something that's … giving you motivational quotes and it’ll just start like repeating them because they only have a few. And then it's just like, okay, I saw this like two days ago and it's just annoying. | Neutral |
|  | P02: I think [the tweet] just has to come across [as] authentic … If it feels like it's just this, fake automated … message system, I'm not going to take it serious. | Neutral |
|  | P12: I think it's fine if people want to [rate the tweets], but I … would not use the thumbs up or thumbs down. I would either just read it and move on or I would snooze it. | Neutral |
|  | P22: I like [the tweets] … if they feel positive, but … like the fourth [tweet] down, I feel like it's like kinda rude. | Neutral |
|  | P24: I think it's not that bad reading [the tweets], but … at the same time … it doesn't look like artificial, doesn't look fake, it looks like actual real people typing … That brings some type of relief. | Positive |
|  | P28: I like the way that [the tweets are] separated into the different types and that you can just … swipe to the right to look through the other ones. That's a lot better than a long list, click each individual thing, to pull up what it says.^1^ | Positive |
|  | P10: The thumbs up and thumbs down makes sense. | Positive |
|  | P30: I think [the tweets are] a good idea. | Positive |
|  | P26: I think that would be nice. I think it looks a lot better [with the tweet poster’s name]. | Positive |
|  | P08: I like the way that [the tweets are presented] now [without names]. | Positive |
|  | P14: I like the way [the tweets page] looks.^1^ | Positive |
|  | P31: I don’t really have a problem with [the tweets page] at all.^1^ | Positive |
|  | P14: It can kind of be a struggle with trying to figure out what would be the best solution to provide that verification to users [that the tweets are authentic] and ensure that it would be a reliable verification source … catering to all walks of life that might be wanting to take advantage of the app because it’s a great idea. | Positive |
|  | P16: No dislikes. I think [the tweets are] great. | Positive |
|  | P08: I like the idea of thumbs up and the thumbs down [for the tweets]. | Positive |
|  | P04: I do agree that it’s cool to know that [the tweets are] like quotes from real people or … people that … already went through it and … want to encourage others. | Positive |
|  | P05: I like the snooze [option for the tweets]. | Positive |
|  | P05: I like how you guys … have quotations around [the tweets] so they know it's something that someone said … maybe … it's an ex-smoker from Twitter or something like that. | Positive |
|  | P11: I think that's awesome, especially the snooze button so you can read [the tweet] later. | Positive |
|  | P13: I like the idea of it being personalized. If it's not just … a tweet but … if it's an actual post and someone is actually talking about it like, I had to go through this and this and I was able to do this. | Positive |
|  | P24: I personally stopped using Twitter, so maybe it would be kinda nice to see what people are still saying … Those tweet formats … they're very blunt or they’re very … funny or sarcastic, but, at the same time, I think … it gives some type of … realness to the app, and it's like a community, somewhat. | Positive |
|  | P13: I like how [the tweet] looks and I like the blue [background], it's kinda calming.^1^ | Positive |
|  | P23: I think [the snooze option for the tweets is] nice. It's just kind of like an alarm clock, sort of … You just kind of turn it on, turn it off. | Positive |
|  | P18: I think that [rating the tweets is] a good idea. | Positive |
|  | P17: Yeah, [rating the tweets is] a good idea. | Positive |

Participant ID appears before each quote for attribution.
FC = Facilitating conditions.
^1^ Indicates quote references design concepts.

**Supplementary Table 4.** **Illustrative quotes of participants’ suggestions for improving a Twitter/X message-based library.**

| **Theme** | **Quotations** |
| --- | --- |
| Content and presentation | P28: Make sure [the tweets are] relevant. |
|  | P37: I think … this could work if someone was like really good at picking tweets … with … a theme that is shared rather than like … random feelings of positive … Because it can all come from different … head spaces. So, I think if it shared … like a gentle theme or something … Nothing like that [first and third example tweet] … I think it would really have to be more of a centralized … feel to the messages, rather than just being everybody's different opinions. |
|  | P36: Yeah, I think the [tweets] that make value judgements against other people … I would want those to be moderated out. |
|  | P35: I like a theme [for the tweets] as well. Not just like random bits, because it does seem a little corny like that. But a theme would be cool. |
|  | P14: I agree, I definitely think you should keep [the tweets] exactly as they are [with spelling and grammatical mistakes] because it also adds an element of it … I know a lot of people are posting statuses and they're not mindful of what it is, or adding apostrophes. And you know they’ll put a space with fragments, and incomplete sentences all the time, but it kind of adds to that realness of what you're reading. |
|  | P16: We use social media a lot, we do a lot of misspelling, we miss punctuations, and all of that stuff. So … if that is gonna be coming from an actual person … you want to show … not just the good, but the ugly too [in the tweets], you know not everyone can spell. |
|  | P13: I think just using Twitter … it sounds like you'd have to go over, like, all the messages and stuff before they’re posted, because … if people aren’t spelling things right, or it's not useful information, or it’s just like people talking … it’d be a bit annoying. |
|  | P20: [The tweets should be as they are with spelling and grammatical errors] because that's what makes it real, because it is real … nothing’s edited, it's not … polished or … fixed up. It’s raw and it's real. It's real and it's a real person … without being coached. |
|  | P20: I think [the tweet feature is] a good idea, but I agree with … filtering [them], because … I wanted it [to] be personable as in … it's real people … as exactly as they put it online with their words, but at the same time … it has validity to it … Like, it's not boring or it's not unnecessary or unrelated … but at the same time … it's modern and real. |
|  | P05: I would say … keep [the tweets] word for word as best as you can unless … there's a lot of spelling errors. If it would get to a point where it would kind of interfere with the understanding of it I might do some brief edits so that … everyone who gets it can understand it. |
|  | P06: I was just saying you should just keep the [tweets] less formal. Make it feel like the messages are from friends. Just keep it less formal. |
|  | P12: If you have a bunch of misspelled tweets, it's kind of … unprofessional and doesn't exactly reflect well … I think it would have to be something kind of curated to really get the best ones on there. |
|  | P21: I think maybe having [the tweets] being … screened first. |
|  | P21: Maybe just doing like a screen through before you choose [tweets] … going through them first and choosing ones that are more geared to positivity than the others. |
|  | P02: As long as [the tweet] comes across authentic … Honestly, I wish there was some kind of link, or I … could connect … just to feel like the person story a little more. But, if you're not going to do that, then just keep it as authentic as possible. |
|  | P11: If you can be selective and find [tweets] that don't have errors that's always best, right. Because … it was kind of unprofessional, and … frustrating, to read something, if everything’s … spelled wrong, but … if you want tweets, then that's … probably what you're gonna get. |
|  | P11: [Spelling and grammatical mistakes should be included in the tweets] probably. That’s about being authentic. |
|  | P24: [The tweets] can say … like mile distance if that's not … creepy to anyone … Like, so and so, from 12 miles away … to make it feel like, okay, they're not in Milwaukee, they’re far away … You could probably like, make someone feel like they're nearby, like someone nearby or … distance wise … don't tell me about everyone who quit, you could maybe help me feel like they're nearby. |
|  | P13: I would hope that you'd pick like pick good [tweets] and as far as like … emojis, I don't really care about those too much. But it would be frustrating trying to read something and you just can't because it's just … all messed up kind of thing. |
|  | P05: I would just be cautious with what [Twitter] messages you use … But I like the idea, like, getting a notification. Maybe … every day or twice a day or something that has something kind of inspirational to help you. But … I think it's … really a matter of choosing ones … that are all positive. |
|  | P23: I think like [spelling and grammatical mistakes] adds character … just … leaving [the tweets] how they are. |
|  | P13: [I would want] a … longer post. Sort of something like on a forum, or maybe like a subreddit … Those are always a lot more helpful and you |
|  | P22: Like simple grammatical things [in the tweets] I would edit just so you feel like … I can trust this person because they know how to spell this word. |
|  | P30: It's just [the tweets] have to be reviewed … before they're actually … put in. |
|  | P24: I think after a while, seeing everything typed up, so perfect kind of takes out … the feeling of it being real, but then possibly seeing emojis or seeing abbreviations [in the tweets] make it relatable. Like someone is actually human like me. |
|  | P22: I think [including spelling and grammatical mistakes in the tweets] would make it authentic as long as it’s readable. |
|  | P08: I think so, [that the tweets should be as they are with spelling and grammatical errors] because … it brings personalization and you know it's from a real person … that's how they're feeling, that's how they're writing. I don't think it should be changed at all. |
|  | P10: It would kind of humanize [the tweets] if there were some emojis and stuff like how they would usually tweet. |
|  | P06: If likes and dislikes are being used to filter [the tweets] … then it's a very good idea, but if they’re just there and you're just collecting my data and you're not using it to … give me recommendations or filter the messages that I’m receiving, then it’s totally useless. |
| Timing | P01: I would say [to receive a tweet] before [a craving]. Maybe it would just help me to get my mind off … it, completely. |
|  | P02: I agree [with receiving a tweet before a craving occurs] … after is cool too, but definitely before. |
|  | P21: I'm actually … trying to think of [whether it would be better to receive a tweet] before or during [a craving]. I don't really know which one … Once … you have a week of … identifying your triggers maybe give the option of … okay, well, you know you logged a lot of your triggers at 5 PM would you like a tailored message before 5 PM or at 5 PM? So, I think if the app could take the information from the user and use that to kind of … gear the questions. Yeah, I think that would be a good idea … I guess maybe having an option that says I'm having a craving, can I have a tip? |
|  | P14: Yeah, definitely [receive a tweet] before [a craving occurs], a while before that even gets close to circulating throughout my thoughts, I think would be beneficial for me. |
|  | P20: I would [want to receive the tweets] definitely mainly before and during [cravings]. But sometimes you don't know when those things are going to occur. But I think it's really important that … you have something right then, because … in the middle of a trigger, you're not going to be thinking, oh, hey, let me do this or that … It like takes your mind off. It lets you take a step back … like, oh, hey, this is a trigger … it kinda puts your mind in gear of where it needs to be. |
|  | P05: I'd [want to receive] say maybe 2 or 3 [tweets a day] … Let's say like morning, afternoon, and evening. Maybe not have a specific time, but … like a time range. Like, between … 8 and 10 and then in between 12 and 2 and 5 and 7 or something like that, just so it’s not … on a schedule, so you know at this time you're going to get a message. |
|  | P05: I'd say either [to receive a tweet] before or during a craving, because if it's after the fact … that could potentially lead to a slip, that you just don't receive anything … I actually think before [the craving] would be best because then you'd have … something positive in your mind when the craving hits rather than just … start craving a cigarette, and then all of a sudden you get something motivational. It can still help, but I think getting it before the craving occurs might get you in a little bit of a better headspace to go at and fight that craving. |
|  | P04: I'd rather [receive a tweet] before [a craving] if possible but … you never know when cravings are going to hit. But if you had like maybe an option that you're like, oh, I'm craving something, let me press this button and maybe get a quote for inspiration, that could be helpful … If after a craving you get some words of inspiration … and be like, hey, it's okay even if you slipped up, then you know we’ll start again. |
|  | P04: Oh, yeah [the timing of the tweets can be random]. That I just randomly get a ping and say, hey, you can do it. |
|  | P11: I think it depends on the [tweet], if it's something that can help with cravings … I would want to know that while I'm having the cravings. So I can … get through it faster … Maybe also one after you … pass the craving, if you're … for sure not going to smoke, then it can be like a good job. And then if you'd like doing up smoking … it can be like, okay … don't beat yourself up … here's something that you can do to … get back on track. |
|  | P12: I would agree with [receiving tweets] before [a craving], like try and kinda nip it in the bud. And then if things don't work out … we're going to try again next time but … not some type of … immediate follow-up type thing, that wouldn't be what I want to see |
|  | P13: [I would prefer to receive tweets] I think before … and during … before the craving to maybe … try to stop it and then … during to help prevent it, but if it does happen it would be cool to have something after, but if it doesn't, I don't know. I wouldn’t want a message I guess … after. |
|  | P11: You have a certain time of day that you've marked to be triggering or location, stuff like that. Maybe one [tweet is] on your phone when you wake up. I think it should be really customizable though so that you can have as many or as few as you want. |
|  | P10: I guess [I would prefer to receive messages] before, or during the craving, because if it comes afterwards, then you probably already smoked one. |
|  | P24: Yeah, like a morning message is good. Maybe an afternoon one, and maybe two in the evening, but nothing about like the encouragement Twitter tweets. That would just kind of be … an afternoon thing, I wouldn't want to see that in the morning. |
|  | P24: Maybe … like a pressure level to have [the tweets sent] when I'm craving, after I … felt the craving disappear … At first I would want it to be vigorous, but then after a while … I wouldn't want that many reminders. |
|  | P22: I feel like I'd want to receive [tweets] in the morning before I'm getting my day going just to get in the right headspace. It might help me. |
|  | P18: [I would want to receive tweets] just like frequently … at least 3 times a day. Like morning, afternoon, and then mid-evening … or late evening. |
|  | P05: Maybe if [receiving the tweets] was even randomized, so you're not just … like, oh, at 8 AM every day I'm gonna get a motivational message. It would just be kind of … when you least expect … you're walking down the street or to the store or something and all of a sudden you get something motivational to lift you up. I think it would be pretty cool. |
|  | P17: [I would want to receive tweets] before and after [cravings]. |
|  | P05: If I could get a [tweet] when I'm about to crave a cigarette to say, hey, do this instead, and then another message to check on me afterwards, to make sure if I'm doing said thing … and then another message afterwards … after the craving’s already done and gone to say, congratulations, good job, you successfully not smoked a cigarette, or … like some type of positive words, like, good job, you did it. |
| Tailoring | P21: I think being, just being able to choose it, like saying, okay, three notifications at 12, 5, and 10 … Then maybe even being able to personalize … if you want a tweet or like a tip or … something like that or just a reminder to check the app. |
|  | P08: I'm a chain smoker, I smoke a lot throughout the day so … I like the idea of me setting up personal times for those [tweets] to pop up, rather than just popping up before a cigarette because it could be popping up 20 minutes and an hour after that and two hours after that … I don't know, that could be an annoyance to me, just because I'm … not thinking about opening the app or trying to open the notification at that time. So … setting a time for myself would be helpful. |
|  | P08: Having apps just send you notifications … every hour or two hours … or even 30 minutes … can get super annoying … I end up … deleting most of those apps that do that, so if there was a way that I could set up a specific timeline when I can get those [tweet] notifications, that would be super helpful. |
|  | P07: I also do like the idea of being able to set your own time of when … you could get those [tweet] notifications … Like maybe you can set it like after meals because a lot of people who smoke right after you eat. It's kinda like the first thing that you do is go and smoke cigarettes. |
|  | P09: I … hope that within the app I can set up a place where I can receive the notifications I want to receive. Not too many … to where I do not want the app on. So, I want to be able to control such a thing. |
|  | P22: [The tweets could be personalized to be] maybe closer to you or … around your age, have similar craving triggers. |
|  | P11: Maybe … if that's like another option, like a way to customize [the tweet feature] … if some people do want to see [the tweet ratings] and some don't. |
|  | P13: I really like when you're able to change anything on an app and customize certain things exactly to your liking, so at … [customizing the] time of day [you would receive tweets] …would be great … You could choose, maybe, based off the calendar kind of thing, as well. |
|  | P20: [Being able to choose how many notifications you receive is important] because even down the road you might not need as many reminders [and tweets] as you did in the beginning, or you might need more later on down the road again because … you're going through something. |
|  | P20: I think that is important that you would get to choose those top things [like how many tweets you receive], because … like some people work at night … some people go to school, some people have kids … So, I think it's important that you would get to choose … what times and how many how many times … you would need it to fit your personal life. |
|  | P11: Finding [tweets] that you want [is important] because … when … it's just like a pop or like a quote … that's just … annoying … But if you can … go … to like a forum or subreddit and … you can seek out the ones that you want to see, that's always … better. |
|  | P21: Yeah, I agree [that you should be able to choose when to receive tweets] … I'm somebody that if an app … bombards me with notifications, I [am] immediately turned off by the app … I don't like that. I don't like that at all. |
|  | P02: I feel like [the tweets are] really personal and probably even could like learn me in a way where if I’m having … a bad day or … there’s certain times where I feel like I need a little more support then … Just to kind of like throw it at me and then if I have to change it then I will change it, you know? |
|  | P14: I do I feel like it'd be nice for users if y'all added … being able to also kind of personalize [the tweets you receive], to an extent, to be able to add in your own messages, if you'd like … Someone can just … pretend that it's some person named Nicole Johnson from Maryland that is writing this testimony. I don't personally have a Twitter account … a lot of people don't have a Facebook or things like that. So, if you were to incorporate a link to that person's profile … I don't know how you would incorporate like a verification element within it, I just can think of options that all kind of have a potential block. |
|  | P02: I like the personalization [suggestion] … It gives me the opportunity to feel like I’m giving them feedback on … whether or not this is appropriate. |
|  | P03: I would like for it to send me different [tweets based on the feedback I provide] … Because when I look at this I think of … Pandora … and there's a song that I don't like, so it skips that song … gives me a different song, right? And then, let's say I liked that song, so it's gonna bounce off of that song, and the artists that I'm looking up underneath … and give me different songs based off of that one song that I liked. |
|  | P20: I think that … if [the tweet ratings are] being used … to … tailor to the next message type of thing, or for like future messages, then I think it's a great idea. |
|  | P21: [Rating the tweets] goes back to the personalization idea … Like the [example tweet about heading outside] … I like that idea. I like being outside, so … might be great for me … Someone else might see them and be like … I don't want to go out. So, I think … tailoring it to the next one. So, maybe the next one I get would be something that says … just go outside and take a few breaths of air. |
|  | P01: I think it would definitely make things better … if when you see [a tweet] that you don't like and you do the thumbs down and it just tries to filter that … that kind of stuff out, then when you give the thumbs up it tries to show you more things related to that topic. |
|  | P11: The rating thing is cool, especially if that … goes back and edits what kind of messages that you … receive. Like, if … you start getting a bunch of … things about drinking more water and you don't like that, then it's going to stop showing you things like that. But if you do like it, it'll show you more. |
| Attribution | P27: I don't see anybody's names [attributed to the tweets] … I can see … states, right at the top, or Mary, I guess that could be added. |
|  | P14: Were you all thinking about implementing like a link … where that post had originated from to kinda validate the source referencing information? … I guess it just depends on where y’all are pulling these from. I don’t know if they’re users … that are posting on to the app itself … or if they're pulled from sources like Twitter or … Instagram and things of that sort … It would be nice … because a lot of them can just be made up by anyone and … just including that maybe at the bottom underneath … the name, or next to the name and … little icon, if that could be a link to view the post source, and then it was a true real user, not a robot. |
|  | P03: I would say … either putting … the person's first name up under [the tweet] … like when they do … the quotes … and put … the little tag thing, and then they put, like, unknown or somethin’ … If it had … at least the first name up underneath it, then … it would make me feel like, okay, well, this came from a real person … Unless … it's saying anonymous or unknown user, or whatever. Like, how do I know somebody didn't just get this out of like, Google, or something. |
|  | P21: Maybe just using their … Twitter handle … Maybe just using their like username and like a small little thing under the tweet. |
|  | P20: Yeah, I can see [adding the Twitter handles] because … I have seen apps or even websites … even advertisement on TV … will have … what someone's put on social media about said product or whatever … and they … put their handle also … Their post … is screenshotted and it shows a little picture … and their handle and then their … text with it. |
|  | P06: Just mention the name of the platform the messages are from but exclude the name of the people who posted. Keep them anonymous. |
|  | P05: [Don’t include] their actual name [of the person who wrote the tweet] though unless you know it's like a celebrity or something. |
|  | P11: If you're going to have [tweets], yeah, it's better to know that it's from a real person than like a computer-generated thing. |
|  | P12: The authenticity [of the tweets] … that's what we want to see, I feel … if you're looking for it [so I would want to know the tweets came from real people]. |
|  | P11: Right after that [tweet], I guess you could say like the name or location or whatever information [of the person who posted it]. |
|  | P13: Like on TV, the [tweets] like pop up [with] your … Twitter box kind of thing. Can do the same thing for the app [and show the user’s Twitter handle]. |
|  | P08: Maybe if you could get people's permission, you can actually get … their Twitter handles, which might be beneficial. |
|  | P13: You can just have [the tweets appear] like … on … TV, where … the tweet on the bottom just comes out with the user's name or something … It just has like the little Twitter box. |
|  | P22: I would think [the tweets would include a name]. “Karen from Maryland said blah, blah, blah,” and that … would personalize it enough for me. |
|  | P07: Obviously, if the people who made those quotes are okay with their names [being included] … then that's great … because then … it kind of gives them a personal touch … It could kinda come from anywhere. I mean, it's more like a reassurance that you know, again, there are people out there that have done it and that you can do it too. |
|  | P17: A real name [attributed to the tweets] would help … I guess it’s fine with the emojis. |
| Frequency | P03: [How many tweets I would want to receive] would … really depend on my mood and stuff and what I got going on for that day, because … if I'm in a good mood and I'm in a good place in my life and stuff … I don't want my phone blowing up every 5, 10 minutes while I’m at work, or whatever, with some random quote … and stuff like that. But … if I was having a bad day … I'd probably want … that extra … quote … or that extra 10 quotes or whatever, for that day. But it just would depend on me and my mood and what I had going on that day. |
|  | P01: During the week, when I work, let's say three [tweets] a day would be plenty, like the first one … when I'm headed to work, about 6:30 in the morning. The next one, when … I'm about to have my cigarette after lunch so at about 12:30, and then finally the last one on my way home at like five. Now, on the weekends, I wouldn't want my phone going off at six in the morning because I definitely wouldn't be out of bed. Yeah, that would be the personalization for me. |
|  | P16: So, in my opinion, it would be that if you're making a really good progress, the less [tweets] that you may need, or it can be the opposite … if you're doing really good, more [tweets] … I don't know. I think maybe start off three a day—morning, noon, night. |
|  | P06: [I would want to receive] like 5 [tweets] a day at random times. That’d be good. |
|  | P14: I wouldn't mind if I got 10 of these [tweets] a day if they were to just pop up on my screen and I could read it and then … touch my phone, and it disappear … If you could add this element into that preference or setting screen option and users could specify as well if … if it didn't cater to a more progress type … that's just my personal preference as far as … outreaching and notifications from all my phone apps, I think the simpler the better. And I love to see stuff like this throughout the day, if it was just a simple sentence that says, hey. It's a reminder … from so and so, and wherever … and show this sentence on my home screen. |
|  | P22: I feel like I'd want to receive … one motivational [tweet] a day. |
|  | P17: [I would want to receive] like 5 [tweets a day]. |
| Additional suggestions | P13: Maybe … people that use [the app] could maybe write stuff that they’re going through and maybe those are … some of that inspirations … that can be posted, instead of Tweets or tips? |
|  | P27: I'm also anticipating … a way where you can communicate with that person [who wrote the tweet], make a connection where they … they can help you in your journey. |
|  | P11: I like that idea [of being able to connect with other users] because then you could … make your own [messages] or even … take someone else's. If you like really connect with a message, you can be like set this as my reminder for like 3:30 PM. |
|  | P37: I think [the tweet feature page] still could, like the other pages kinda, just use more color? |
|  | P34: It'd be cool if [the tweet feature] was … like a social media in itself, to where you're talking to other people using app, going through your journey, and then you guys share your own messages back and forth and whatnot. |
|  | P11: I think if [the tweet feature page] had … fun pictures or something because then … like this sharable thing … you could post that on … your Instagram story or whatever. |
|  | P11: I think having options [for the tweet feature], like turn it off if you don't want to see it would be good. |
|  | P03: Let's say somebody … wrote an inspirational quote or had … a real deep, deep story or something? Is there a way that we could kinda … click on them and … talk to them to be like, hey, that was really deep, I liked what you said, how did you do it, or you know? … Like a … chat place … to where we could talk to other smokers to get advice … or get help from them … to like kinda bounce off of each other type thing … Like to have people to … help you … or that you could help and stuff like that. |
|  | P13: I think it would be helpful if it actually showed the rating [of the tweets] as well. So, basically people can thumbs up, you know, this helped me. So, you know it helped other people … it might help you as well kind of thing or, oh no, this is just useless information kind of thing. It would be nice to not only be able to rate it [but] to see the ratings. |
|  | P16: I think that maybe there should be … a section where people can share their stories. Because a lot of times … I'll watch a lot of stuff and … like a lot of times I think cancer of smoking and I have kids. So … that scares me but it's like, again, I can't stop smoking. So, I think … people sharing their stories, and being able to go and just see what people go through, and what smoking is causing for them … I think that'll be … a good section in the app … just to see … this is reality for a lot of people, this is the reality for smokers. So, just to get others opinion and their life stories, I think that'll be great if that can be a section for the users. |
|  | P11: I think not seeing the ratings [for the tweets] would be better, just cause … if I hit thumbs up, and everyone else hit thumbs down, then I'm gonna feel … silly … I feel like that can make people feel ostracized. |
|  | P03: My only thing would be … [to be] able to … get a support group or something … I think that would really be helpful … if like people was able to click on [the tweet] and talk to the person that wrote that … I think that would be really helpful and useful as well. |

Participant ID appears before each quote for attribution.
